# Physical Activity and Sedentary Time: Association with Metabolic Health and Liver Fat

**DOI:** 10.1249/MSS.0000000000001901

**Published:** 2019-01-25

**Authors:** KELLY A. BOWDEN DAVIES, VICTORIA S. SPRUNG, JULIETTE A. NORMAN, ANDREW THOMPSON, KATIE L. MITCHELL, JO A. HARROLD, GRAHAM FINLAYSON, CATHERINE GIBBONS, JOHN P. H. WILDING, GRAHAM J. KEMP, MARK HAMER, DANIEL J. CUTHBERTSON

**Affiliations:** 1 Institute of Ageing and Chronic Disease, University of Liverpool, Liverpool, UNITED KINGDOM;; 2 School of Biomedical Sciences, Faculty of Medical Sciences, Newcastle University, Newcastle upon Tyne, UNITED KINGDOM;; 3 Obesity and Endocrinology Research Group, Clinical Sciences Centre, University Hospital Aintree, Liverpool, UNITED KINGDOM;; 4 Research Institute for Sport and Exercise Science, Liverpool John Moores University, Liverpool, UNITED KINGDOM;; 5 Wolfson Centre for Personalised Medicine, Institute of Translational Medicine, Liverpool, UNITED KINGDOM;; 6 Department of Psychological Sciences, Institute of Psychology Health and Society, University of Liverpool, Liverpool, UNITED KINGDOM;; 7 Appetite Control and Energy Balance Research, School of Psychology, Faculty of Medicine and Health, University of Leeds, Leeds, UNITED KINGDOM;; 8 Liverpool Magnetic Resonance Imaging Centre (LiMRIC), University of Liverpool, Liverpool, UNITED KINGDOM; and; 9 School Sport, Exercise Health Sciences, National Centre for Sport and Exercise Medicine-East Midlands, Loughborough University, Loughborough, UNITED KINGDOM

**Keywords:** BODY COMPOSITION, MAGNETIC RESONANCE SPECTROSCOPY, METABOLIC SYNDROME, INSULIN REGULATION, CARDIORESPIRATORY FITNESS, METABOLIC EQUIVALENTS

## Abstract

**Introduction/Purpose:**

To investigate whether (a) lower levels of daily physical activity (PA) and greater sedentary time accounted for contrasting metabolic phenotypes (higher liver fat/presence of metabolic syndrome [METS+] vs lower liver fat/absence of metabolic syndrome [METS−]) in individuals of similar body mass index and (b) the association of sedentary time on metabolic health and liver fat.

**Methods:**

Ninety-eight habitually active participants (53 female, 45 male; age, 39 ± 13 yr; body mass index 26.9 ± 5.1 kg·m^−2^), underwent assessments of PA (SenseWear armband; wear time ~98%), cardiorespiratory fitness (V˙O_2_ peak), body composition (magnetic resonance imaging and magnetic resonance spectroscopy) and multiorgan insulin sensitivity (oral glucose tolerance test). We undertook a) cross-sectional analysis comparing four groups: nonobese or obese, with and without metabolic syndrome (METS+ vs METS−) and b) univariate and multivariate regression for sedentary time and other levels of PA in relation to liver fat.

**Results:**

Light, moderate, and vigorous PA did not account for differences in metabolic health between individuals, whether nonobese or obese, although METS+ individuals were more sedentary, with a higher number, and prolonged bouts (~1–2 h). Overall, sedentary time, average daily METS and V˙O_2_ peak were each independently associated with liver fat percentage. Each additional hour of daily sedentary time was associated with a 1.15% (95% confidence interval, 1.14%–1.50%) higher liver fat content.

**Conclusions:**

Greater sedentary time, independent of other levels of PA, is associated with being metabolically unhealthy; even in habitually active people, lesser sedentary time, and higher cardiorespiratory fitness and average daily METS is associated with lower liver fat.

Strong epidemiologic evidence suggests an inverse relationship between physical activity (PA) levels and obesity, metabolic syndrome (METS), nonalcoholic fatty liver disease (NAFLD) and type 2 diabetes (1–5). Increased PA is recommended both for individuals and at a population level to improve metabolic health and help prevent these interrelated conditions. The independent protective effect of high cardiorespiratory fitness (CRF), an objective marker of PA, against all-cause mortality is well established (6,7). There is a growing recognition that sedentary behavior, which has an independent association with adverse health outcomes, should be minimized (2,8,9). Increasing moderate PA is protective against the aforementioned diseases and attenuates, but does not eliminate, the detrimental effects of sedentary behavior (10). Breaking up prolonged periods of sedentary time (11) or replacing it with low-intensity PA (12) are beneficial for glycemic control.

Obesity is strongly associated with poor cardiometabolic health and overall mortality (13). However, not all obese individuals are *metabolically unhealthy* (METS+) (14); conversely not all nonobese individuals are *metabolically healthy* (METS−) (15). Some studies suggest that METS+ may be a consequence of low PA (16,17), but others have not supported this conclusion (18–20). With differences in methodology, cohort characteristics and definitions of metabolic phenotypes, these studies typically have not precisely defined the differences in PA characteristics between phenotypes. Only one study, of older adults, has *objectively* measured sedentary behavior (19), which offers better reliability than self-report (21); no such study has been undertaken in young to middle-age adults. There are similarly conflicting results in studies of the association of metabolic health with objectively measured sedentary behavior and quantitative measures of liver fat using magnetic resonance spectroscopy (MRS) or computed tomography (22–26). The accumulation of liver fat has been described as a major contributor to the development of type 2 diabetes (27) and is considered the hepatic manifestation of METS and closely linked with obesity and insulin resistance (28). Observing levels of PA, including sedentary behavior, in metabolic phenotypes of a given body mass index (BMI) category with further quantification of liver fat may reveal associations which link habitual activity to health outcomes and the predisposition for metabolic diseases.

This cross-sectional study will objectively monitor the habitual PA of young to middle-age adults and extensively phenotype these individuals by assessment of metabolic health and magnetic resonance imaging (MRI)-derived body composition. We hypothesize that greater sedentary time and lower levels of PA will be evident in metabolically unhealthy phenotypes (METS+ vs METS−) in BMI-matched individuals; and second, higher MRS-quantified liver fat will be associated with greater sedentary time and lower PA levels.

## METHODS

### Participants

Habitually active individuals, who engaged in no more than 2 h of exercise per week, were recruited via local advertisements across University of Liverpool campuses and hospital departments. Exclusions included cardiovascular, respiratory, kidney, liver and/or endocrine complications, smoking, and >14 units per week of alcohol consumption. The study conformed to the *Declaration of Helsinki* and was approved by the North West Liverpool Central research ethics committee (14/NW/1145; 14/NW/1147; 15/NW/0550). All participants were informed of the methods verbally and in writing before providing written informed consent before any assessments. Ninety-eight individuals (52 male, 46 female) with a mean age of 39 ± 13 yr and BMI of 27 ± 5 kg·m^−2^ were recruited. Before each study visit, participants were required to fast overnight for 12 h (water was permitted *ad libitum*), abstain from alcohol and caffeine for 24 h and from exercise for 48 h.

### Study Design

All participants completed measurement of baseline PA and dietary consumption over a period of 4 d (including one weekend day) between January 2016 and February 2017 followed by assessment in the order of (a) anthropometry (including bioimpedance), fasting biochemistry, an oral glucose tolerance test, and assessment of CRF (V˙O_2_ peak) at University Hospital Aintree and (b) MRI and proton MRS (^1^H-MRS) at the University of Liverpool MRI Center. Because of the MRI scanner replacement during part of this study, MRI quantification of body fat was conducted in only 72 individuals. Bioimpedance data were collected in all individuals, and V˙O_2_ peak calculations were based on both total body mass and fat-free mass (FFM).

### Individual Phenotyping

Individuals were characterized into one of four groups based on BMI (nonobese, <30 kg·m^−2^ vs obese, ≥30 kg·m^−2^) and the presence or absence of METS according to International Diabetes Federation criteria; we refer to these groups as (i) “nonobese METS−,” (ii) “nonobese METS+,” (iii) “obese METS−” and (iv) “obese METS+.”

### Habitual Assessment

#### PA monitoring

Physical activity was monitored throughout using a validated (29) SenseWear mini armband (BodyMedia Inc., Pittsburgh, PA). Wear time (recorded as ~98%) was monitored using SenseWear Professional software (version 8.0). Data included the following: daily average step count, total energy expenditure, active energy expenditure, and time spent in levels of PA including: sleep, lying down, sedentary (<1.5 METS), light (1.5–3 METS), moderate (3–6 METS), vigorous (6–9 METS), and very vigorous (>9 METS). A Microsoft Excel template, as previously described (30), was used to determine how sedentary time (not including sleep) was accumulated and provided information on the frequency of bouts and the time accumulated in a given bout category (<1 h: 1–5, 6–10, 11–20, 21–40, 41–60 min; 1–2 h: 61–80, 81–100, 101–120 min; >2 h: 121–140, 141–160, 161–180 min). To examine “frequently broken” periods of sedentary time, the given bout categories at the lower end (<1 h) were shorter in duration. At the higher end (>1 h), where fewer bouts are recorded, the given bout categories are greater in duration. Based on previous observations (31), this approach was adopted to investigate “patterns” of sedentary time, that is, the frequency with which sedentary time is interrupted (sedentary breaks) or the duration of uninterrupted periods of sedentary time (sedentary bouts). Furthermore, moderate to vigorous PA (MVPA) of bouts greater or less than 10 min were determined.

#### Dietary analysis

Total energy consumption, carbohydrate, protein, and fat content were determined from 4-d dietary records by a registered nutritionist (K.M.) using Nutritics (Nutrition Analysis Software for Professionals; https://www.nutritics.com/p/home).

### Other Assessment Measures

#### Anthropometric measurements

Stature (Model 220, Seca, Germany) and whole-body bioimpedance analysis (Tanita, BC 420, Dolby Medical Stirling, UK) was conducted; this provided total body mass, fat percentage, fat mass, FFM, muscle mass, total body water, basal metabolic rate, bone mass, and visceral fat indicator. Waist and hip circumference measurements were taken in duplicate, and blood pressure was determined from an average of three measures (Dinamap, G & E Medical, USA).

#### Biochemical measurements

Blood samples were collected and analyzed using the Olympus AU2700 analyzer (Beckman Coulter, High Wycombe, UK) with standard proprietary reagents as follows: glucose with hexokinase, total cholesterol and high-density lipoprotein with cholesterol esterase/oxidase and triacylglycerol with glycerol kinase. Low-density lipoprotein was calculated according to the Friedwald formula. Insulin was measured using radio-immunoassay (Invitrogen, UK). HOMA-IR was calculated using fasting glucose and insulin concentrations.

#### Oral Glucose Tolerance Test

Following a 12-h fast, blood samples were collected, a 75-g glucose drink was consumed within 5 min and postingestion blood samples were drawn at 30, 60, 90, and 120 min. Matsuda index was calculated to estimate whole-body IS, and indices of hepatic-IR and skeletal muscle IS were determined as previously described (32).

#### CRF

A V˙O_2_ peak cardiopulmonary exercise test was performed on a treadmill (Model 77OCE; RAM Medisoft Group, Manchester, UK) in a temperature-controlled room. The cardiopulmonary exercise test provided breath-by-breath monitoring and analysis of expiratory gases and ventilation (Love Medical Cardiopulmonary Diagnostics, Manchester, UK). The modified Bruce protocol was employed, after an initial 2-min warm-up at 2.2 km·h^−1^ on a flat gradient, stepwise increments in speed and gradient were employed each minute. V˙O_2_ peak was determined by exhaustion plus one or more of: respiratory exchange ratio >1.15, heart rate >90% predicted maximum, plateau in V˙O_2_.

#### ^1^H-MRS

Liver and skeletal muscle fat were determined using a 1.5T Siemens Symphony MRI scanner as previously described (33).

### Statistical Analysis

All data were explored for normality using visual inspection of frequency distribution, and logarithmically transformed where appropriate. Given the small sample size, power achieved on each test was assessed and ranged from 46% to >99%; 20 of 26 achieved at least 80% power. Age was analyzed using a one factor between-groups ANOVA, whereby a significant group effect was observed (*P* < 0.05). Between-group univariate general linear models were conducted for all other variables, with age as a covariate and Bonferroni correction for multiple comparisons. Statistically significant interactions were explored, and nominal *P* values reported. Univariate and multivariate linear regressions were used to analyze components of PA and fitness associated with liver fat. Decisions were made *a priori* to include all variables reaching *P* < 0.1 in univariate regression analysis alongside age and BMI in the multivariate regression model. The statistical cutoff for inclusion in the final model is more stringent than often used to guard against false discovery. The alpha level of statistical significance was set at *P* < 0.05. Data are presented as mean (95% confidence interval), unless stated otherwise. Transformed data were back-transformed to original units. *P* value >1 rounded to 1.000.

## RESULTS

### Participant Characteristics

The numbers of individuals with each risk factor of METS are summarized in Table 1, with the PA and CRF data of the whole cohort combined. Calculated from their average of 4-d MVPA (accumulated in bouts of >10 min), 61% of individuals met the World Health Organization recommendations.

### Metabolic Phenotyping

The significant differences between the groups’ components of METS were in line with International Diabetes Federation classification (Table 2). There was no significant difference between obese METS− and obese METS+ BMI (*P* = 0.712) but nonobese METS+ BMI was 3 ± 2 kg·m^−2^ greater than nonobese METS− (*P* = 0.003). In the general population, MRS defined that liver fat >5.5% corresponds with the prevalence of hepatic steatosis (34); 84 and 14 participants had liver fat <5.5% and ≥5.5%, respectively.

### Dietary Intake

Total energy consumption, carbohydrate, protein and fat did not differ significantly between groups (*P* > 0.05). Mean ± SD macronutrient percentages were 56% ± 16% carbohydrate, 24% ± 9% protein, and 20% ± 7% fat.

### CRF

**FIGURE 1:**
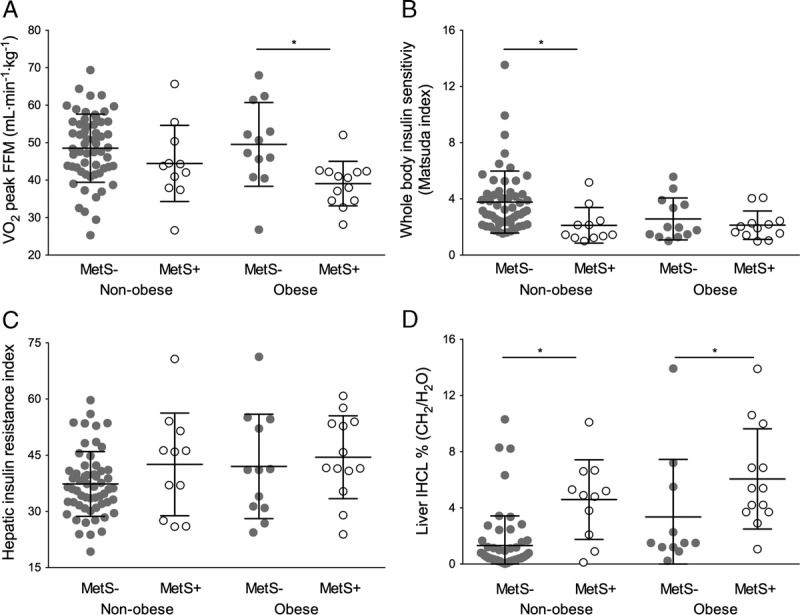
Cardiometabolic phenotyping, individual participant plots for: V˙O_2_ peak relative to FFM (A), whole-body insulin sensitivity (B), hepatic insulin resistance index (C) and liver intrahepatocellular lipid (IHCL) (D). Data are presented as mean ± SD. *Gray circles*, METS−; white circles, METS+; nonobese are grouped left and obese are grouped right. *P < 0.05 group difference between BMI category, further group differences being given in the text.

Obese METS+ individuals had lower CRF than both obese and nonobese METS− (*P* ≤ 0.029; mean difference ≥7.5 mL·min^−1^·kg^−1^) but not nonobese METS+ (*P* = 0.675; mean difference 5.9 mL·min^−1^·kg^−1^) There was no difference between both nonobese groups and obese METS− (*P* ≥ 0.080) (Fig. 1A).

### Multiorgan IS

Nonobese METS− individuals had greater Matsuda index than nonobese METS+ (*P* = 0.012; mean difference, 2.0) (Fig. 1B); there was no difference between obese METS− and both METS+ groups (*P* ≥ 0.141). There was no group effect for skeletal muscle IS index (*P* = 0.220). Hepatic-IR index was greater in obese METS+ than nonobese METS− (Fig. 1C). There was a significant group effect (*P* = 0.022) for HOMA-IR.

### MRS Quantification of Liver Fat

Liver fat was higher in METS+ in both nonobese and obese. Nonobese METS− individuals had 4.6% lower liver fat than obese METS+ (*P* ≤ 0.005) (Fig. 1D). Liver fat percentage in nonobese METS+ was not different to either obese group (*P* ≥ 0.794; mean difference, ≥0.6%); and liver fat percentage in obese groups was not statistically different (*P* = 0.336; mean difference, 2.6%).

### Levels of PA: Differences between the Four Metabolic Phenotypes

#### Average daily steps

**FIGURE 2:**
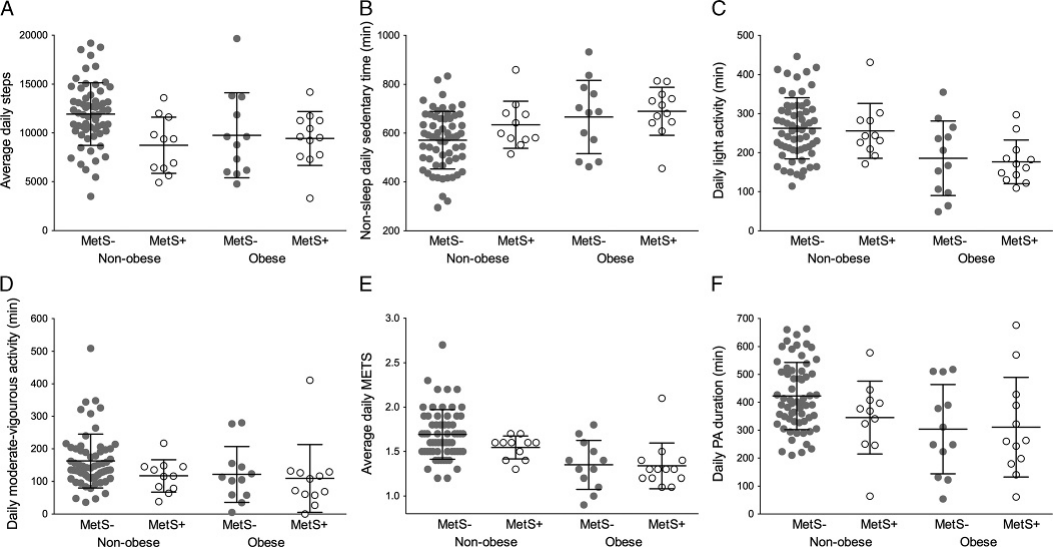
Habitual PA and sedentary time, individual participant plots for: average daily steps (A), nonsleep sedentary time (<1.5 METS) (B), light activity (1.5–3 METS) (C), moderate to vigorous activity (>3 METS) (D), daily metabolic equivalents (METS) (E) and PA duration (F). Data are presented as mean ± SD. Gray circles, METS−; *white circles*, METS+; nonobese are grouped left and obese are grouped right. *P < 0.05 group difference between BMI category, further group differences being given in the text.

There was no group effect for average daily steps (Fig. 2A).

#### Nonsleep sedentary time, lying time, and sleep duration

Nonsleep sedentary time (Fig. 2B) was not different between nonobese groups (*P* = 1.000; 49 min·d^−1^·) and obese groups (*P* = 1.000; 23 min·d^−1^·). Nonobese METS− individuals had lower sedentary time than obese METS+ (*P* = 0.04); there was no difference between obese METS− and both METS+ groups (*P* ≥ 0.199). There was no group effect for amount of time spent lying down (*P* = 0.080) or sleeping (*P* = 0.117).

#### Daily light PA time

There was no difference in daily light activity between both nonobese groups (*P* = 0.711; mean difference, 10 min·d^−1^) and both obese groups (*P* = 1.000; 9 min·d^−1^). However, both obese groups had less light activity than both nonobese METS− (*P* ≤ 0.015; mean difference ≥ 69 min·d^−1^) (Fig. 2C).

#### Daily MVPA time

There was no difference between the groups’ moderate to vigorous activity (*P* = 0.322) (Fig. 2D), and no significant differences were found for the way in which MVPA was accumulated for bouts of 10 min or more, in either total minutes accumulated or percentage of the time in relation to total MVPA.

#### Average daily METS and PA duration

Daily average METS (Fig. 2E) and PA duration (Fig. 2F) had significant group effects (*P* < 0.0005 and *P* = 0.020, respectively); for both measures, nonobese METS− had greater values than both obese groups, but were not different to nonobese METS+. Daily average METS in nonobese METS− were 0.3 METS greater than both obese groups (*P* < 0.0005). The same was observed for PA duration, with nonobese METS− having greater duration that both obese groups (*P* ≤ 0.018; mean difference ≥107 min·d^−1^). There was no significant difference between obese METS− and both METS+ groups for average daily METS and PA duration (*P* ≥ 0.079 and *P* ≥ 0.450, respectively).

#### Patterns of waking sedentary time

**FIGURE 3:**
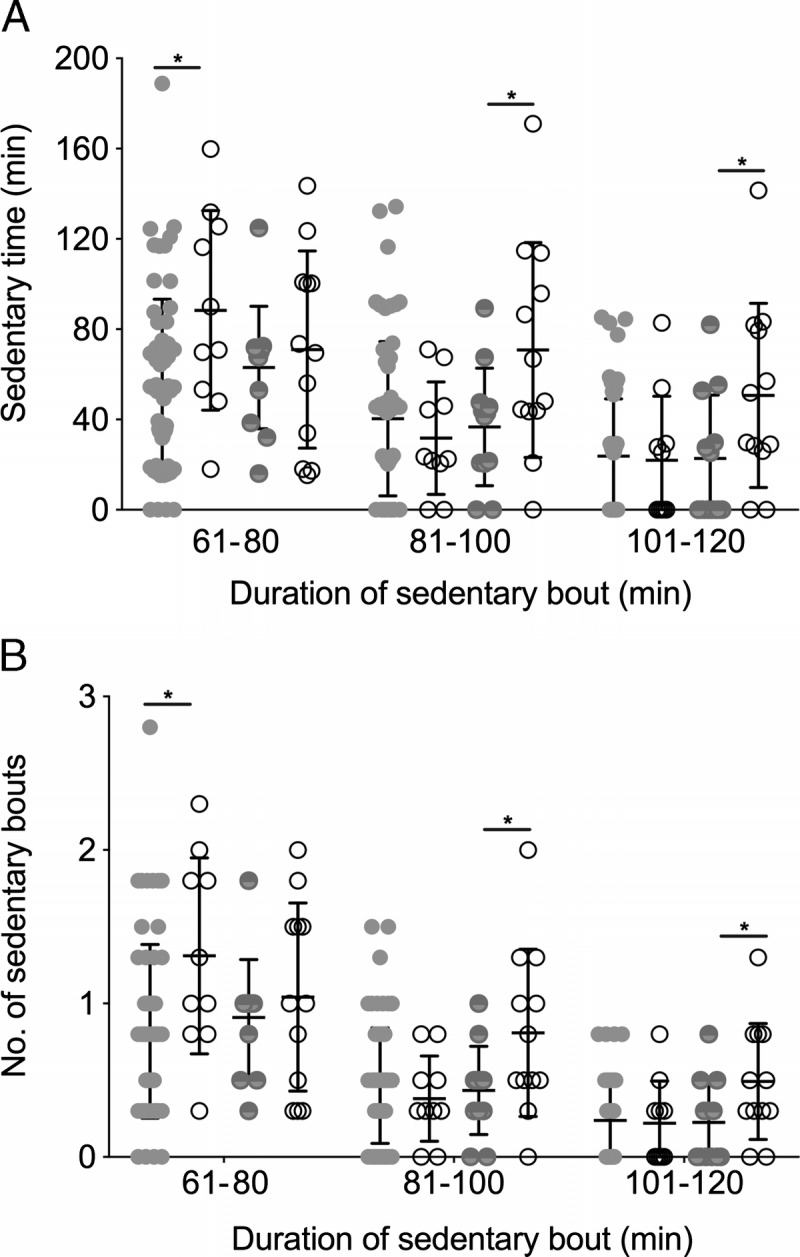
Nonsleep sedentary behavior, individual participant plots for: duration of sedentary bouts (A) and number of sedentary bouts in given bout category (B) between 1 and 2 h. Data are presented as mean ± SD. *Gray circles*, METS−; *white circles*, METS+; nonobese are grouped left and obese are grouped right. *P < 0.05 group difference between BMI category, further group differences being given in the text.

Analysis of sedentary behavior was performed on waking sedentary time examining the *duration of sedentary time* (Fig. 3A) and *the number of sedentary bouts* (Fig. 3B) in a predetermined bout category. There were no differences between the groups in sedentary bout durations of <1 h or >2 h. However, significant differences were apparent during bout durations lasting between 1 and 2 h.

**TABLE 1:**
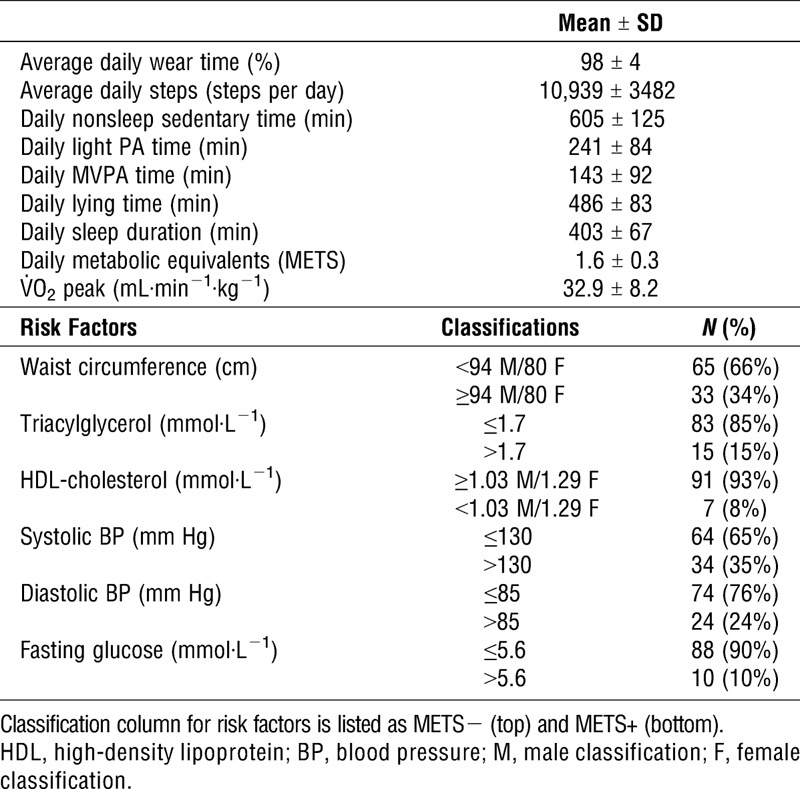
PA and CRF data, the number of risk factors of METS and liver fat in 98 individuals.

**TABLE 2:**
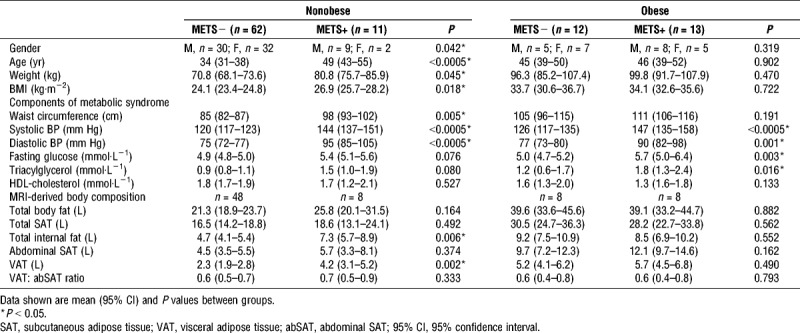
Clinical, metabolic, and body composition characteristics of participants categorized for obesity and subsequently according to METS.

#### Duration

During bouts of 61 to 80 min, nonobese METS+ accumulated 33 min more sedentary time per day than nonobese METS− (3, 60; *P* = 0.013). During bouts of 81 to 100 min, METS+ obese accumulated 34 min·d^−1^ more than obese METS− (6, 62; *P* = 0.018). During bouts of 101 to 120 min, obese METS+ accumulated 28 min·d^−1^ more than obese METS− (5, 51; *P* = 0.018).

#### Number of bouts

As an average of 4 d, both METS+ groups accumulated one to two more long bouts (between 1 and 2 h) of sedentary behavior, compared with their METS− counterparts. Considering bouts of 61 to 80 min, nonobese METS+ had 0.5 more bouts per day (0.1–0.9; *P* = 0.012) than METS−. Obese METS+ had 0.4 more bouts per day (0.1–0.7; *P* = 0.019) than METS− of 81 to 100 min and 0.3 more bouts per day (0.1–0.5; *P* = 0.017) of 101 to 120 min.

#### Levels of PA (regression analysis)

Univariable linear regression analysis revealed that daily average steps, sedentary time, vigorous activity, METS, and V˙O_2_ peak were all significantly associated with liver fat. Carried forward in the multivariable analysis, three of these factors remained statistically significant predictors of liver fat (Table 3). Greater daily sedentary time is associated with higher liver fat, while higher overall daily METS and V˙O_2_ peak are associated with lower liver fat (Fig. 3). For a 1-h increase in sedentary time, liver fat increased by 1.15% (1.14%–1.50%; *P =* 0.036), whereas for a 1-mL·min^−1^·kg^−1^ increase in CRF (V˙O_2_ peak), liver fat reduced by 0.87% (0.25–1.50; *P* = 0.007).

**TABLE 3:**
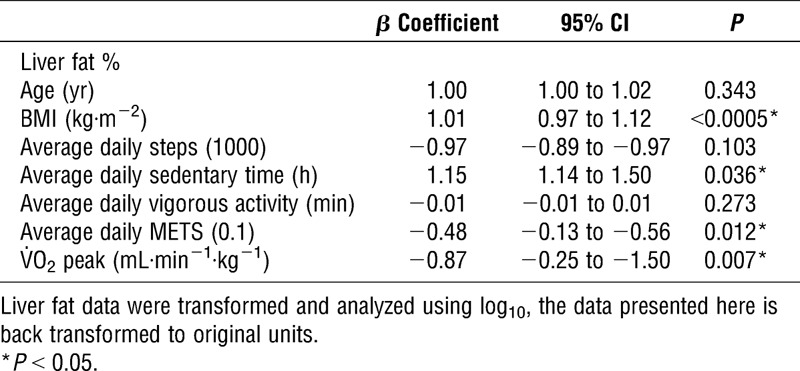
Multivariate regression for liver fat percentage (%).

## DISCUSSION

The results of this extensive phenotypic analysis of objective measurements of PA and sedentary behavior, metabolic and body composition measurements (including MRS-derived liver fat) in young-middle age adults demonstrate two key messages. First, in this cohort, overall habitual PA was not associated with different metabolic health status in individuals of similar BMI, and the accumulation of sedentary time was weakly associated with the presence of the METS. Second, even in habitually active individuals, there is an association between greater sedentary time and increased liver fat, whereas the amount of MVPA appeared to have little independent association. These data highlight the potential importance of sedentary behavior in determining optimal metabolic health and liver fat.

It is recognized that greater sedentary time increases the risk of becoming overweight/obese (35) and the risk of type 2 diabetes and cardiovascular disease, even after controlling for MVPA (8,36). Although total volumes of habitual PA do not explain metabolic health in this cohort, those with METS shown some evidence of being more sedentary, with a higher number of prolonged bouts of sedentary behavior (between 1 and 2 h). Frequent breaks in sedentary time have been shown to be beneficial to metabolic risk (31), health (37) and liver fat (24). To our knowledge, there are no studies which have investigated sedentary bouts greater than 1 h. Interestingly, an extra hour of sedentary time has been associated with a 39% increased odds for METS (38) and decreasing sedentary time accumulated in prolonged bouts may have beneficial effects on BMI and waist circumference (39). Further research at durations of >1 h may reveal insight into the pattern in which sedentary time is accumulated and METS. Even individuals who are physically active can still spend a significant amount of their waking day sedentary (termed previously as “sedentary exercisers” (40)), which is associated with increased cardiometabolic risk. Taken together, these findings suggest that public health and chronic disease prevention strategies that largely focus on MVPA recommendations might benefit from new recommendations regarding interruption of prolonged sedentary time, complimentary to those of PA.

Numerous prospective studies have confirmed the relationship between PA and liver fat (5,41–44) and compliance with national MVPA guidelines has been associated with a lower odds of NAFLD (26). Furthermore, recent research in a population-based sample of adults has shown that V˙O_2_ peak is strongly, inversely, and independently related to the risk of liver fat (45). The results presented are in agreement with previous research, greater levels of PA (here daily METS) and higher CRF is independently associated with lower levels of liver fat. Importantly, the associations between CRF and liver fat remained after adjustment for BMI; not all studies have reported similar findings (46). The association between sedentary time and liver fat is equivocal. Some authors have found no associations between PA or sedentary behavior and liver fat in 82 individuals (25,26). Whereas others have concluded that PA and sedentary time are indeed independently associated with the prevalence of NAFLD (22,24). In *inactive* individuals, every hour of sedentary time was associated with increases of 1.74 L of total abdominal fat, 0.62 L of visceral fat, 1.14 L of subcutaneous fat, and 1.86% liver fat (22). Direct comparisons or broad conclusions are difficult due to differences in cohorts and methodology. The findings of the current study suggest that sedentary time has an independent effect on liver fat in active adults; however, more data are required to confirm this. Our results, demonstrating that every hour of additional sedentary time translates to a 1.15% increase in liver fat, can be put into context by comparing the effects of a 4-wk aerobic cycling intervention in sedentary obese men and women, where liver fat reduced by 1.7% (47). The effects surgical, nutritional, lifestyle, or pharmaceutical interventions aiming to reduce liver fat has been recently reviewed (48).

This study uses objective monitoring of PA, gold standard measurement of CRF and MRS-derived liver fat in young-middle age adults, all of which are key strengths. The results did not support any strong evidence for a beneficial association of sedentary bouts <1 h or detrimental association of >2 h perhaps due to study limitations which include the relatively small sample size. Further limitations include: duration of PA assessment, the monitor used to assess sedentary behavior (SenseWear does not determine postural differences), the comparatively healthy habitual PA habits of the participants which somewhat limits the external validity of the findings, and the cross-sectional design which cannot determine causality. Noteworthy is the higher BMI in unhealthy nonobese versus healthy nonobese which conforms to the association of a greater BMI with greater metabolic risk. This difference could not be controlled for because it was a component of our grouping analysis but differences in age were statistically controlled for. Although the present results demonstrate that overall sedentary time needs to be considered independently of PA, objective PA monitoring in a larger cohort with a prospective design will be required, and future research should further explore sedentary behavior patterns (i.e., amount of sedentary breaks and duration of sedentary bouts). The American Diabetes Association has recommended that adults should “decrease the amount of time spent daily in sedentary behavior” and that “prolonged sitting should be interrupted with bouts of light activity every 30 min.” Importantly, these recommendations are in addition to, not a substitute for, a physically active lifestyle. A “cutoff” for harmful sedentary behavior patterns (i.e., frequency/duration) has not been defined in public health guidelines.

In summary, in habitually active adults, the amount of sedentary time is associated in this single-measure observation with metabolic health and the quantity of liver fat. The findings of this study highlight that public health policy designed to optimize the benefits of PA may need to synergistically consider strategies to reduce sedentary behavior.
